# Characterization, Antioxidant Capacity, and Anti-Inflammatory Activity of Polyphenol-Enriched Extracts Obtained from Unripe, Mature, and Overripe Fruits of Red-Fleshed Kiwifruit Cultivars

**DOI:** 10.3390/foods13182860

**Published:** 2024-09-10

**Authors:** Qian-Ni Yang, Wen Deng, Ding-Tao Wu, Jie Li, Hong-Yan Liu, Hui-Ling Yan, Kui Du, Yi-Chen Hu, Liang Zou, Jing-Wei Huang

**Affiliations:** 1 Key Laboratory of Coarse Cereal Processing of Ministry of Agriculture and Rural Affairs, School of Food and Biological Engineering, Chengdu University, Chengdu 610106, China; 2 Institute for Advanced Study, Chengdu University, Chengdu 610106, China; 3 Research Center for Plants and Human Health, Institute of Urban Agriculture, Chinese Academy of Agricultural Sciences, National Agricultural Science and Technology Center, Chengdu 610213, China; 4 China-New Zealand Belt and Road Joint Laboratory on Kiwifruit, Kiwifruit Breeding and Utilization Key Laboratory of Sichuan Province, Sichuan Provincial Academy of Natural Resource Sciences, Chengdu 610015, China

**Keywords:** thinned unripe kiwifruits, polyphenols, antioxidant capacity, anti-diabetic activity, anti-inflammatory activity

## Abstract

Discarded unripe kiwifruits (DUKs) are regarded as the major agro-byproducts in the production of kiwifruits, which have abundantly valuable secondary metabolites. Nevertheless, owing to the limited knowledge about the differences in phytochemicals and bioactivity between DUKs and mature kiwifruits, the utilization of DUKs in the food industry remains scarce. Hence, to promote their food applications, the phenolic compounds and bioactivity of discarded unripe, mature, and overripe fruits from three red-fleshed kiwifruit cultivars were studied and compared. The results revealed that the levels of total phenolics, total flavonoids, and total procyanidins in kiwifruits varied significantly by maturity stage. In addition, our findings demonstrated that DUKs possessed much higher contents of valuable phenolic compounds (e.g., chlorogenic acid (CHA), neochlorogenic acid (NCHA), gallic acid (GA), protocatechuic acid (PA), procyanidin B1 (ProcB1), procyanidin B2 (ProcB2), procyanidin C1 (ProcC1), quercetin 3-O-glucoside (QueG), and quercetin 3-O-rhamnoside (QueR)) than mature and overripe kiwifruits. Furthermore, DUKs exerted much stronger in vitro antioxidant capacity, inhibitory effects on α-glucosidase, and anti-inflammatory activity than mature and overripe kiwifruits, which were mainly attributed to their higher contents of total polyphenols and individual phenolic components, such as GA, CHA, NCHA, PA, ProcB1, ProcB2, ProcC1, and QueR. Overall, these findings provide sufficient evidence for the development and utilization of DUKs in the food/functional food industry.

## 1. Introduction

Kiwifruit belongs to the genus *Actinidia* in the family *Actinidiaceae*. It is a nutrient-rich and bioactive compound-laden food, and is highly regarded for its delicious taste [1]. At present, China, Italy, New Zealand, Greece, and Chile are the major producers around the world, accounting for about 87% of the total production of kiwifruit [1]. To date, about seventy-five kiwifruit species have been recognized around the world, exhibiting a wide range of skin appearance and flesh color [1]. In fact, kiwifruits can be categorized into green-fleshed, yellow-fleshed, and red-fleshed cultivars based on their flesh colors [1]. Indeed, ‘Hayward’ has been regarded as the leading green-fleshed kiwifruit cultivar in the world, and the common cultivars of yellow-fleshed kiwifruit are ‘Hort-16’, ‘Jinyan’, and ‘Jinshi’ in New Zealand, Italy, and China [2,3]. Different red-fleshed cultivars (e.g., ‘Donghong’, ‘Hongyang’, ‘Hongshi’, and ‘Qihong’) are mainly grown in China, including in Shaanxi Province, Sichuan Province, Guizhou Province, Hunan Province, Hubei Province, and Henan Province [2,3].

Generally, the consumption of red-fleshed kiwifruits can be a good choice for the amelioration and prevention of oxidative damage and chronic metabolic syndrome owing to their strong antioxidant capacity. Polyphenols are regarded as one of the most important bioactive components in kiwifruits, which can contribute to their numerous biological activities, e.g., antioxidant, anti-inflammatory, anti-diabetic, anti-tumor, anti-ulcer, hypoglycemic, and hypolipidemic effects [1,4,5]. In particular, several studies have revealed that red-fleshed kiwifruits possess more valuable polyphenols than green-fleshed and yellow-fleshed cultivars, exhibiting much stronger antioxidant capacity [2,3,6].

Usually, to achieve superior fruit quality for customers, fruit thinning is commonly carried out by orchard workers, and more than thirty percent of unripe kiwifruits will be removed at the early growing stage (20–60 days after fruit-setting) [7,8]. These thinned unripe kiwifruits are considered as the major agro-byproducts in the production of kiwifruits, which are usually discarded in orchards, causing serious environmental pollution and resource wasting [8]. Therefore, suitable management or processing of these discarded unripe kiwifruits (DUKs) is required to promote their potential applications. In fact, recent studies have revealed that discarded unripe red-fleshed kiwifruits are rich in bioactive ingredients, e.g., phenolic compounds and pectic polysaccharides, which possess various health-promoting benefits [7,8]. In addition, an earlier study also showed that discarded unripe fruits from green-fleshed and yellow-fleshed kiwifruit cultivars contain much higher contents of phenolic compounds than mature fruits, thereby exhibiting superior antioxidant capacity [9]. These results suggest that DUKs have good potential to be utilized in the common food and functional food sectors.

Nevertheless, knowledge regarding the differences in bioactive polyphenols between discarded unripe fruits and mature fruits from red-fleshed kiwifruit cultivars is still limited, which ultimately restricts their potential applications. Therefore, to promote their development and application in the food or functional food industry, the phenolic compounds and health-promoting effects of discarded unripe fruits (20 days after fruit-setting, YK stage), mature fruits (110 days after fruit-setting, MK1 stage), and overmature fruits (170 days after fruit-setting, MK2 stage) from three red-fleshed kiwifruit cultivars (*A. chinensis* cv. ‘Hongao’, ‘Hongshi’, and ‘Hongyang’) were investigated and compared. The findings can provide good evidence for the application of discarded unripe fruits from red-fleshed kiwifruit cultivars in the food and functional food industries.

## 2. Materials and Methods

### 2.1. Chemicals and Reagents

Three red-fleshed kiwifruit cultivars, including *A. chinensis* cv. ‘Hongao’, *A. chinensis* cv. ‘Hongshi’, and *A. chinensis* cv. ‘Hongyang’, were collected from Deyang kiwifruit breeding and planting base, Sichuan, China. In detail, discarded unripe kiwifruits (DUKs) were collected at 20 days after fruit-setting (YK stage). Mature and overmature kiwifruits were harvested at 110 days after fruit-setting (MK1 stage) and 170 days after fruit-setting (MK2 stage), respectively.

Phenolic standards, including gallic acid (GA), ferulic acid (FA), caffeic acid (CA), *p*-coumaric acid (*p*-CA), chlorogenic acid (CHA), neochlorogenic acid (NCHA), protocatechuic acid (PA), catechin (CN), epicatechin (ECN), procyanidin B1 (ProcB1), procyanidin B2 (ProcB2), procyanidin C1 (ProcC1), quercetin 3-O-glucoside (QueG), and quercetin 3-O-rhamnoside (QueR) were purchased from Shanghai Yuanye Biotechnology Co., Ltd. (Shanghai, China). Acarbose tablets were obtained from Bayer HealthCare Co., Ltd. (Beijing, China). Interleukin-6 (IL-6) and tumor necrosis factor-α (TNF-α) ELISA kits were purchased from Wuhan Elabscience Biotechnology Co., Ltd. (Wuhan, China). The nitric oxide assay kit was purchased from Shanghai Beyotime (Shanghai, China). All other chemicals used were of analytical grade.

### 2.2. Preparation of Polyphenol-Enriched Extracts and Determination of Total Polyphenols

Polyphenol-enriched extracts from unripe, mature, and overripe kiwifruits were prepared following our previously established ultrasound-assisted deep eutectic solvent extraction (UADEE) method [8]. Polyphenol-enriched extracts from ‘Hongao’ (HA), ‘Hongshi’ (HS), and ‘Hongyang’ (HY) kiwifruit cultivars harvested at three different stages, including the YK stage, MK1 stage, and MK2 stage, were coded as HA-YK, HS-YK, HY-YK, HA-MK1, HS-MK1, HY-MK1, HA-MK2, HS-MK2, and HY-MK2, respectively. After the UADEE extraction, the levels of total polyphenols, including total phenolic content (TPC), total flavonoid content (TFC), and total procyanidin content (TPAC), were detected by colorimetric methods according to a previous study [8]. The levels of TPC, TFC, and TPAC in different polyphenol-enriched extracts are expressed as mg GAE/g DW, mg RE/g DW, and mg CE/g DW, respectively. Detailed information regarding the extraction conditions and different colorimetric methods is supplied in the Supplementary Materials (Sections S.1 and S.2).

### 2.3. Qualitative Analysis of Phenolic Compounds in Different Polyphenol-Enriched Extracts by LC-Q-TOF-MS Analysis

Various phenolic compounds in polyphenol-enriched extracts obtained from unripe, mature, and overripe kiwifruits were characterized by high-resolution LC-Q-TOF-MS (Agilent 6545 Q-TOF-MS, Agilent Technologies, Santa Clara, CA, USA) following a previously reported method with minor modifications [10]. Data analysis was carried out using Agilent Qualitative Analysis 10.0 software and Agilent PCDL Manager B. 08.00 software. Extracted ions were cross-referenced with those from the literature and databases (TCM-database, Agilent Technologies, Santa Clara, CA, USA) as well as some authentic phenolic standards to identify phenolic compounds in different polyphenol-enriched extracts. Detailed information regarding the LC-Q-TOF-MS analysis is supplied in the Supplementary Materials (Section S.3).

### 2.4. HPLC Analysis of Phenolic Components in Different Polyphenol-Enriched Extracts

The major phenolic components in polyphenol-enriched extracts obtained from unripe, mature, and overripe kiwifruits were detected by HPLC analysis following a previously established method [11]. Fourteen commercial standards, including six phenolic acids (GA, CA, *p*-CA, FA, CHA, and NCHA), six flavanols (PA, CN, ECN, ProcB1, ProcB2, and ProcC1), and two flavonols (QueG and QueR), were quantified, and their calibration data are shown in Table S1. The level of each phenolic compound is expressed as μg/g DW. More detailed information regarding the HPLC analysis is suppled in the Supplementary Materials (Section S.4).

### 2.5. Assessment of Antioxidant Capacity of Different Polyphenol-Enriched Extracts

To systematically understand the differences in the antioxidant capacity of polyphenol-enriched extracts obtained from unripe, mature, and overripe kiwifruits, three different assays (ABTS, DPPH, and hydroxyl radical (OH) radical scavenging ability assays) were carried out following previously established methods [8,11]. The IC_50_ values of different polyphenol-enriched extracts against ABTS, DPPH, and OH free radicals are expressed as milligram kiwifruit dry weight per milliliter (mg/mL). More detailed methods for the assessment of antioxidant capacity of different polyphenol-enriched extracts are suppled in the Supplementary Materials (Section S.5).

### 2.6. Assessment of Inhibitory Effect of Different Polyphenol-Enriched Extracts against A-Glucosidase

To understand the differences in the potential anti-diabetic effect of polyphenol-enriched extracts obtained from unripe, mature, and overripe kiwifruits, their inhibitory effect against α-glucosidase was measured following a previously established method [8]. An acarbose tablet was applied as a positive control, and the IC_50_ values of different polyphenol-enriched extracts against α-glucosidase are expressed as μg/mL. More detailed methods for the assessment of the inhibitory effect of different polyphenol-enriched extracts against α-glucosidase are also suppled in the Supplementary Materials (Section S.5).

### 2.7. Assessment of Anti-Inflammatory Activity of Different Polyphenol-Enriched Extracts

To understand the differences in the potential anti-inflammatory activity of polyphenol-enriched extracts obtained from unripe, mature, and overripe kiwifruits, the lipopolysaccharide (LPS)-induced RAW 264.7 cell model method was carried out according to a previous study with minor modifications [8]. The cytotoxic effect of each sample on RAW 264.7 cells was measured, and then the inhibitory effect of each sample on the release of proinflammatory factors from LPS-induced RAW 264.7 cells, including NO, IL-6, and TNF-α, were measured by different kits following the manufacturers’ protocols. More detailed methods are also suppled in the Supplementary Materials (Section S.5).

### 2.8. Statistical Analysis

A one-way analysis of variance or two-tailed Student’s t-test was carried out for statistical analysis by using Origin 2022 software (OriginLab Co., Northampton, MA, USA), and statistical significance was set at *p* < 0.05. Hierarchical cluster heatmap analysis was carried out to explain differences and similarities among different kiwifruits. Additionally, Pearson correlation coefficients were measured using Origin 2022 software (OriginLab Co., Northampton, MA, USA).

## 3. Results and Discussion

### 3.1. Comparison of TPC, TFC, and TPAC in Unripe, Mature, and Overripe Fruits of Three Red-Fleshed Kiwifruit Cultivars

**Figure 1:**
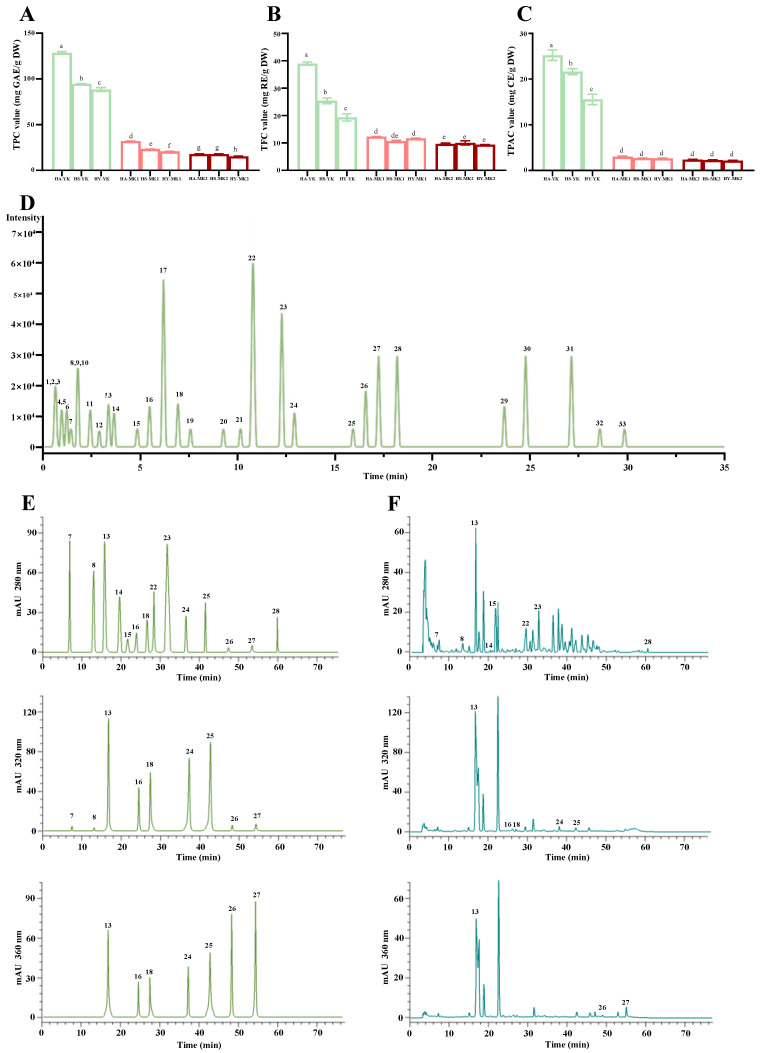
Total phenolics (**A**), total flavonoids (**B**), total procyanidins (**C**), LC-Q-TOF-MS extracted ions chromatogram (**D**), and HPLC profiles of mixed phenolic standards (**E**) and the presentative extract of discarded unripe kiwifruits (**F**). HA-YK, HS-YK, HY-YK, HA-MK1, HS-MK1, HY-MK1, HA-MK2, HS-MK2, and HY-MK2 indicate polyphenol-enriched extracts obtained from ‘Hongao’ (HA), ‘Hongshi’ (HS), and ‘Hongyang’ (HY) kiwifruit cultivars harvested at different maturation stages, including the immature stage (YK), mature stage (MK1), and overmature stage (MK2), respectively; compounds 1–33 are the same as in Table 1. Different lowercase letters (a–h) indicate statistically significant differences among different unripe, mature, and overripe kiwifruits (*p* < 0.05).

A recent experimental result has demonstrated that discarded unripe red-fleshed kiwifruits are rich in valuable polyphenols [8]. Nevertheless, systematic knowledge about the variations of bioactive polyphenols between discarded unripe and mature fruits of different red-fleshed kiwifruit cultivars is still limited, which ultimately restricts their potential applications. To promote their potential food applications, the levels of TPC, TFC, and TPAC in polyphenol-enriched extracts obtained from unripe, mature, and overripe kiwifruits were studied. Figure 1A–C summarize the levels of total polyphenols in different polyphenol-enriched extracts. The findings showed that the levels of TPC, TFC, and TPAC varied greatly by different maturity stages and cultivars, which were comparable to earlier studies which showed that both cultivar and maturation stage of kiwifruits had remarkable impacts on their levels of total polyphenols [12]. Notably, the levels of total polyphenols exhibited a drastic decreasing pattern during fruit maturation, especially during the early stage of growth. In detail, the levels of TPC in different DUKs ranged from 88.45 (HY-YK) to 128.39 mg GAE/g DW (HA-YK), which were much higher than those of different mature and overripe kiwifruits, ranging from 20.63 (HY-MK1) to 31.89 mg GAE/g DW (HA-MK1), and from 15.31 (HY-MK2) to 17.75 mg GAE/g DW (HA-MK2/HS-MK2), respectively. In addition, as shown in Figure 1A–C, the changing trends of TFC and TPAC in different unripe, mature, and overripe kiwifruits were consistent with that of TPC. These findings implied that maturation stage, especially the early stage of fruit growing, exerted strong impacts on the levels of total polyphenols in red-fleshed kiwifruit cultivars, which were comparable to earlier studies [9,13,14]. In addition, apart from maturation stage, cultivar also had a dramatic impact on the contents of TPC, TFC, and TPAC in red-fleshed kiwifruit cultivars at the early stage of growth, with the highest contents of TPC, TFC, and TPAC observed in discarded ‘Hongao’ unripe kiwifruits (HA-YK), followed by ‘Hongshi’ and ‘Hongyang’. This phenomenon was comparable to earlier studies which showed that total polyphenols in kiwifruits at the early growing stage varied significantly by different species and cultivars [9,14]. Furthermore, the average level of TPC in different DUKs was also significantly higher than that of different mature kiwifruits of *A. chinensis* and *A. deliciosa* according to previous experimental studies [9,11,15], with the levels in the range of 3.75–16.52 mg GAE/g DW. Overall, these findings clearly demonstrate that DUKs from three red-fleshed kiwifruit cultivars are potential sources of valuable polyphenols, and can provide sufficient evidence for the development and application of DUKs in the food industry.

### 3.2. Comparison of Major Phenolic Compounds in Unripe, Mature, and Overripe Fruits of Three Red-Fleshed Kiwifruit Cultivars

Polyphenolics, including phenolic acids (e.g., hydroxybenzoic acids and hydroxycinnamic acids), flavanols (e.g., catechin derivatives and polymeric procyanidins), flavonols (e.g., quercetin derivatives and kaempferol derivatives), and anthocyanins (e.g., cyanidin derivatives and delphinidin derivatives), are commonly observed in kiwifruits of different species and cultivars [4,12,16,17]. Nevertheless, the variations of phenolic compounds in discarded unripe fruits and mature fruits from different red-fleshed kiwifruit cultivars are still unclear. Therefore, the major phenolic compounds in different unripe, mature, and overripe fruits of three red-fleshed kiwifruit cultivars were characterized by high-resolution LC-Q-TOF-MS analysis. As displayed in Figure 1D, thirty-three compounds were tentatively identified, and their detailed information is summarized in Table 1. Obviously, these compounds varied by cultivar and maturation stage, and were comparable to those found in previous studies [9,18]. Specifically, several compounds, e.g., quercetin-3-O-robinobioside, isovitexin, and *p*-coumaric acid-hexose, were only observed in DUKs of three red-fleshed cultivars, and several compounds, e.g., quinic acid, malic acid, citric acid, GA, protocatechuic acid-O-hexoside, cinnamic acid, vanillic acid, CHA, syringic acid, 3-p-coumaroylquinic acid, caffeic acid-O-hexoside, apigenin, and kaempferol, were absent in overripe kiwifruits of three red-fleshed cultivars. In addition, several compounds, e.g., CA, *p*-CA, FA, and QG, were only absent in the ‘Hongyang’ cultivar. Nevertheless, among these thirty-three compounds, most phenolic compounds, e.g., vanillic acid, syringic acid, cinnamic acid, GA, CA, FA, *p*-CA, CHA, NCHA, PA, CN, ECN, ProcB1, ProcB2, ProcC1, QueG, QueR, quercetin, and kaempferol, were commonly observed in mature kiwifruits of different species and cultivars [4,5,12,16,17].

Furthermore, to comprehend the variations in individual phenolic compounds in different unripe, mature, and overripe kiwifruits, their levels were determined by HPLC-DAD analysis. In this study, based on the results of LC-Q-TOF-MS analysis and previous literature [8,11], 14 commercially available phenolic standards were measured. In detail, six phenolic acids (GA, NCHA, CHA, CA, FA, and p-CA), six flavan-3-ols (CN, ECN, PA, ProcB1, ProcB2, and ProcC1), and two flavonols (QueG and QueR), were assessed in all samples. Figure 1E,F display HPLC chromatograms of the mixed phenolic standards and the presentative sample (HS-YK), and their levels are summarized in Table 2. The levels of major phenolic compounds significantly varied by maturation stage, with the highest average content observed in the YK stage (21,967.1 μg/g DW) and the lowest average level observed in the MK2 stage (2842.7 μg/g DW). Obviously, these results displayed that the levels of major phenolic components in different kiwifruits exhibited a dramatic decreasing trend during fruit growth and maturation, which was comparable to the variation pattern of total polyphenols in discarded unripe, mature, and overripe kiwifruits as displayed in Figure 1A–C. These results further confirmed that maturation stage, especially the early growing stage, had strong influences on the levels of polyphenols in kiwifruits, which were similar to earlier studies [9,13,14]. In addition, the levels of major phenolic compounds in kiwifruits at the same growing stage also varied greatly in the three red-fleshed cultivars, with levels ranging from 14,265.0 (HY-YK) to 27,918.0 μg/g DW (HA-YK) at the YK stage, from 2934.8 (HY-MK1) to 5137.1 μg/g DW (HA-MK1) at the MK1 stage, and from 2031.5 (HY-MK2) to 3522.9 μg/g DW (HA-MK2) at the MK2 stage, similar to earlier studies [9,14].

As displayed in Table 2, flavanols were measured as the predominate phenolic compounds in different unripe, mature, and overripe kiwifruits, similar to previous studies [2,8,9]. Although the levels of total flavanols decreased as kiwifruits matured, this study clearly demonstrated that ProcB2, ProcB1, and ECN were observed as the predominant flavanols, and even the predominant phenolic compounds, in all discarded unripe, mature, and overripe kiwifruits. Their levels were also dependent on the cultivar and maturation stage of the kiwifruits. In detail, the levels of ProcB2 in three red-fleshed kiwifruit cultivars ranged from 6459.2 to 8583.1 μg/g DW at the YK stage, from 1563.4 to 1933.3 μg/g DW at the MK1 stage, and from 1242.5 to 1742.2 μg/g DW at the MK2 stage. In addition, the highest content of ProcB1 was found in the ‘Hongyang’ cultivar at the YK stage (3708.5 μg/g DW), while the lowest content was found in the ‘Hongshi’ cultivar at the MK2 stage (155.9 μg/g DW). The highest content of ECN was found in the ‘Hongao’ cultivar at the YK stage (5388.7 μg/g DW), while only minor contents of ECN were determined in the ‘Hongyang’ cultivar at both the YK (210.6 μg/g DW) and MK2 stages (70.3 μg/g DW). In fact, previous studies have also shown that both immature and mature kiwifruits possess abundant ECN, ProcB1, and ProcB2 [2,8,9,11]. Furthermore, compared with ECN, ProcB1, and ProcB2, only small amounts of PA and ProcC1 were observed in all discarded unripe, mature, and overripe kiwifruits, with the levels ranging from 16.6 to 1168.4 μg/g DW and from 29.3 to 418.3 μg/g DW, respectively. Small amounts of CN were observed in both the ‘Hongao’ and ‘Hongshi’ cultivars at different stages. Overall, these results showed that DUKs were rich in flavanols, implying that DUKs could be good sources of phenolic compounds.

As displayed in Table 2, apart from flavanols, phenolic acids composed the second major group of phenolic compounds in discarded unripe, mature, and overripe kiwifruits, and their contents, once again, also varied by cultivar and maturity. Their levels drastically decreased from the YK stage to the MK1 stage, and then gradually declined from the MK1 stage to the MK2 stage, revealing that the early growing stage had strong impacts on the levels of phenolic acids in different kiwifruits [9]. Notably, NCHA was observed as the predominant phenolic acid in different unripe, mature, and overripe kiwifruits, with levels ranging from 1157.4 to 5929.3 μg/g DW at the YK stage, from 207.1 to 382.5 μg/g DW at the MK1 stage, and from 146.9 to 200.7 μg/g DW at the MK2 stage. Previous studies also revealed that both red-fleshed immature and mature kiwifruits were rich in NCHA [8,11,15]. In addition, GA was the second most abundant phenolic acid observed in three kiwifruit cultivars at the YK stage, with levels ranging from 622.8 to 707.7 μg/g DW, which dramatically decreased at the MK1 stage and were even not detectable at the MK2 stage, similar to earlier studies [9,14]. The variation trend of CHA was similar to that of GA. The average levels of CHA also notably declined from the YK stage (172.7 μg/g DW) to the MK1 stage (6.8 μg/g DW), and were even undetectable at the MK2 stage, which was comparable to a previous study which showed that levels of CHA significantly decreased from the immature stage to the mature stage [9]. Furthermore, compared with NCHA and GA, only minor amounts of CA, *p*-CA, and FA were observed in the ‘Hongao’ and ‘Hongshi’ cultivars at different maturity stages, while they were absent in the ‘Hongyang’ cultivar. Overall, these results implied that different unripe kiwifruits, especially the ‘Hongao’ and ‘Hongshi’ cultivars, possessed abundant phenolic acids, thereby exhibiting great potential to be utilized as natural antioxidants.

Two flavonols, including QueG and QueR, were observed in all tested samples, once again, which also varied greatly by cultivar and maturity. The highest average levels of QueG (810.0 μg/g DW) and QueR (614.4 μg/g DW) in kiwifruits were observed at the YK stage, while the lowest levels of QueG (8.1 μg/g DW) and QueR (7.7 μg/g DW) in kiwifruits were seen at the MK2 stage. These results implied that the levels of flavonols in kiwifruits also exerted a drastic decrease from the YK stage to MK1 stage, which was comparable to earlier studies [9,14]. In addition, QueG was not found in the ‘Hongyang’ cultivar at different harvested stages, which was comparable to earlier studies which showed that the cultivar was the important factor affecting the types and levels of flavonols in kiwifruits [2,11,18].

**Figure 2:**
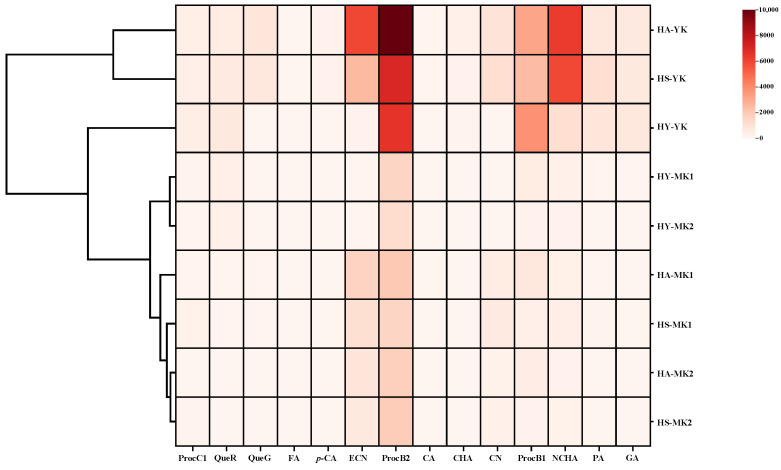
Hierarchical cluster analysis of phenolic compounds in polyphenol-enriched extracts obtained from different discarded unripe, mature, and overripe kiwifruits. HA-YK, HS-YK, HY-YK, HA-MK1, HS-MK1, HY-MK1, HA-MK2, HS-MK2, and HY-MK2 indicate polyphenol-enriched extracts obtained from ‘Hongao’ (HA), ‘Hongshi’ (HS), and ‘Hongyang’ (HY) kiwifruit cultivars harvested at different maturation stages, including the immature stage (YK), mature stage (MK1), and overmature stage (MK2); GA, gallic acid; PA, protocatechuic acid; NCHA, neochlorogenic acid; ProcB1, procyanidin B1; CN, catechin; CHA, chlorogenic acid; CA, caffeic acid; ProcB2, procyanidin B2; ECN, epicatechin; FA, ferulic acid; *p*-CA, *p*-coumaric acid; QueG, quercetin 3-O-glucoside; QueR, quercetin 3-orhamnoside; ProcC1, procyanidin C1.

Moreover, a hierarchical cluster analysis (HCA) was conducted to view the differences and similarities among three kiwifruit cultivars at different harvested stages in terms of their phenolic compounds. As displayed in Figure 2, three distinctive clusters (cluster 1, cluster 2, and cluster 3) could be identified by HCA analysis. The ‘Hongao’ and ‘Hongshi’ cultivars harvested at the YK stage were in cluster 1, which was characterized by greatly higher levels of ECN, ProcB2, ProcB1, and NCHA. Cluster 2 was only composed of the ‘Hongyang’ cultivar harvested at the YK stage, and was characterized by much higher contents of ProcB2 and NCHA. Furthermore, the ‘Hongao’, ‘Hongshi’, and ‘Hongyang’ cultivars harvested at the MK1 and MK2 stages were in cluster 3, which was characterized by lower levels of phenolic compounds. These results further confirmed that both maturation stage and kiwifruit cultivar exerted important impacts on their phenolic compounds, and also provided sufficient evidence for the application of these discarded unripe kiwifruits as promising sources of valuable polyphenols.

### 3.3. Comparison of Antioxidant Capacity of Polyphenol-Enriched Extracts Obtained from Unripe, Mature, and Overripe Fruits of Red-Fleshed Kiwifruit Cultivars

Numerous studies have suggested that the health-promoting effects of the dietary intake of kiwifruits are mainly attributed to their excellent antioxidant capacity that can protect cells from excessive oxidative damage because kiwifruits contain a high level of antioxidants such as phenolic acids, flavanols, flavonols, and anthocyanins [12,16]. Lots of studies have demonstrated that kiwifruits and their extracts exert great antioxidant capacity by scavenging various free radicals, e.g., the ABTS radical, DPPH radical, hydroxyl radical, and superoxide radical [12]. Nevertheless, systematic knowledge about the variations of antioxidant capacity between discarded unripe and mature fruits of different red-fleshed kiwifruit cultivars is still limited. Therefore, to exploit the utilizations of discarded unripe red-fleshed kiwifruits in the food industry, the free radical scavenging abilities of discarded unripe, mature, and overripe fruits of three red-fleshed kiwifruit cultivars were studied and compared.

**Figure 3:**
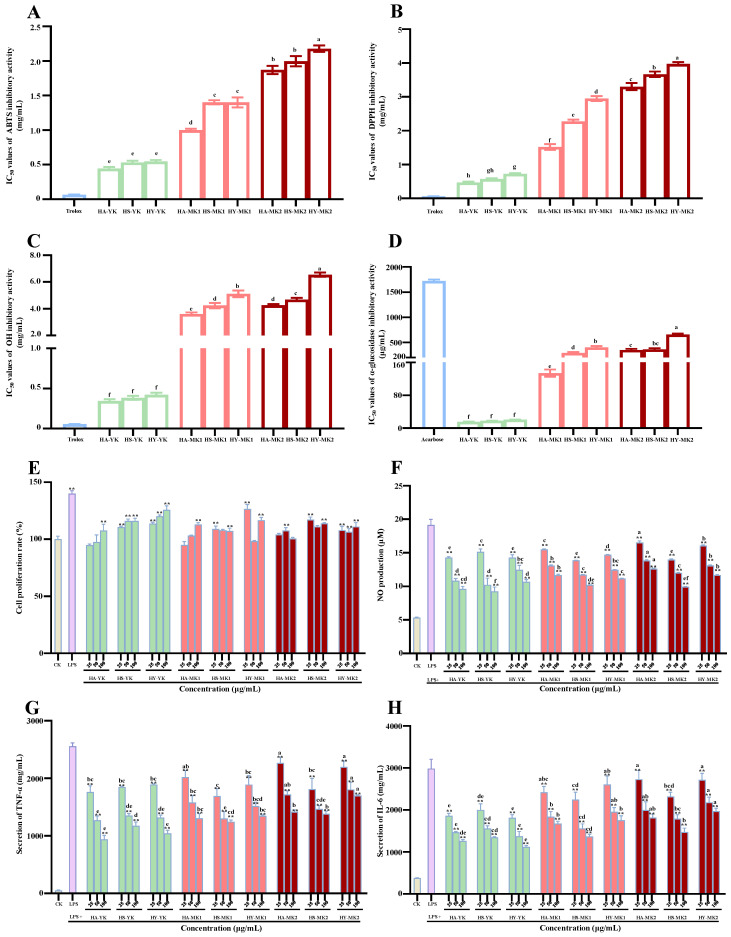
Antioxidant capacity (**A**–**C**), inhibitory effect on α-glucosidase (**D**), and anti-inflammatory activity (**E**–**H**) of polyphenol-enriched extracts obtained from different discarded unripe, mature, and overripe kiwifruits. (**A**), ABTS radical scavenging activity; (**B**), DPPH radical scavenging activity; (**C**), OH radical scavenging activity; (**E**), cell viability of RAW 264.7 macrophages; (**F**–**H**), the release of NO, TNF-α, and IL-6 from LPS-induced RAW 264.7 macrophages. HA-YK, HS-YK, HY-YK, HA-MK1, HS-MK1, HY-MK1, HA-MK2, HS-MK2, and HY-MK2 indicate polyphenol-enriched extracts obtained from ‘Hongao’ (HA), ‘Hongshi’ (HS), and ‘Hongyang’ (HY) kiwifruit cultivars harvested at different maturation stages, including the immature stage (YK), mature stage (MK1), and overmature stage (MK2); different letters (a–h) indicate statistically significant differences (*p* < 0.05) among different discarded unripe, mature, and overripe kiwifruits; significant differences in the cell viability of LPS and kiwifruit extracts vs. control are shown by ** *p* < 0.01. Significant differences in NO production, secretion of IL-6, and secretion of TNF-α in kiwifruit extracts vs. LPS are shown by ** *p* < 0.01.

**Figure 4:**
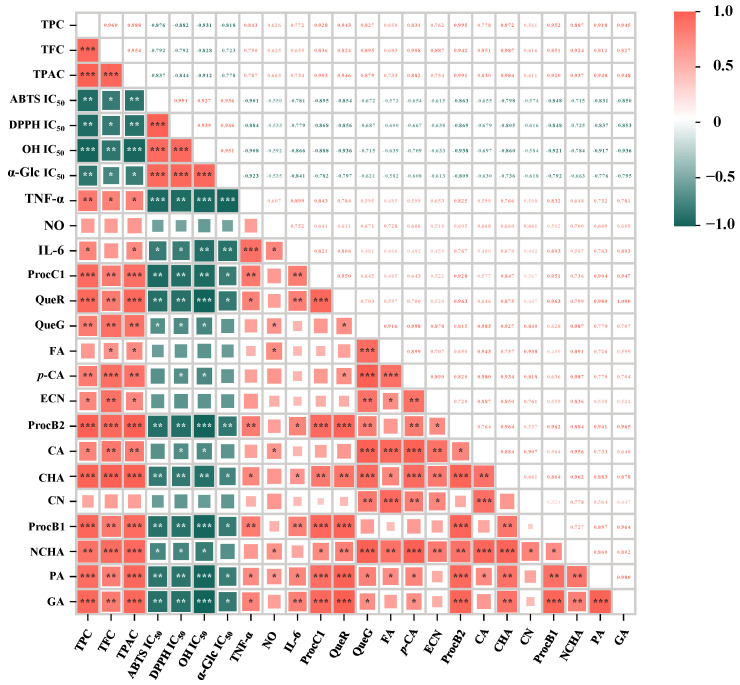
Pearson correlation matrix for total polyphenols, individual phenolics, antioxidant capacity, inhibitory effect on α-glucosidase, and anti-inflammatory activity. TPC, total phenolic content; TFC, total flavonoid content; TPAC, total procyanidin content; GA, gallic acid; PA, protocatechuic acid; NCHA, neochlorogenic acid; ProcB1, procyanidin B1; CN, catechin; CHA, chlorogenic acid; CA, caffeic acid; ProcB2, procyanidin B2; ECN, epicatechin; FA, ferulic acid; p-CA, p-coumaric acid; QueG, quercetin 3-O-glucoside; QueR, quercetin 3-orhamnoside; ProcC1, procyanidin C1; ABTS IC_50_, DPPH IC_50_, and OH IC_50_ indicate IC_50_ values of ABTS, DPPH, and OH radical scavenging activity, respectively; α-Glc IC_50_ indicates IC_50_ values for the inhibition of α-glucosidase; NO, IL-6, and TNF-α indicate NO, IL-6, and TNF-α produced from LPS-stimulated RAW 264.7 cells, respectively; Significant correlations are shown by * *p* < 0.05, ** *p* < 0.01, and *** *p* < 0.001.

As displayed in Figure 3A–C, discarded unripe, mature, and overripe kiwifruits exhibited excellent scavenging abilities against ABTS, DPPH, and OH radicals. Obviously, their scavenging abilities varied greatly between the immature and mature stages, and were comparable to the variation trends of their total polyphenols (Figure 1A) and total phenolic compounds (Table 2). In detail, the results highlighted a significant variation in the antioxidant capacity of different kiwifruits harvested at the YK, MK1, and MK2 stages, with the order being YK > MK1 > MK2. The average IC_50_ values for scavenging ABTS radicals of three red-fleshed kiwifruit cultivars harvested at the YK, MK1, and MK2 stages were measured to be 0.50 mg/mL, 1.25 mg/mL, and 2.01 mg/mL, respectively, and it could be clearly observed that the ABTS radical scavenging ability of unripe kiwifruits was about 2.5 and 4.02 times stronger than that of mature and overripe kiwifruits, respectively. In addition, it could be also observed that the DPPH radical scavenging ability of unripe kiwifruits was about 3.75 and 6.1 times stronger than that of mature and overripe kiwifruits, respectively. The OH radical scavenging ability of unripe kiwifruits was about 11.2 and 13.6 times stronger than that of mature and overripe kiwifruits, respectively. Taken together, DUKs exerted extremely stronger antioxidant capacity than mature and overripe kiwifruits, and maturation stage was proven to be the leading factor for regulating their antioxidant capacity. Similar studies have revealed that immature fruits possess higher antioxidant capacity than mature fruits, such as for green- and yellow-fleshed kiwifruits, peaches, apples, and pomegranates [9,19,20,21]. Furthermore, as displayed in Figure 4, based on the correlation coefficients calculated from the Pearson analysis, the IC_50_ values for scavenging ABTS, DPPH, and OH radicals exerted significantly negative correlations with the contents of TPC (r, −0.931–−0.876), TFC (r, −0.828–−0.792), and TPAC (r, −0.912–−0.837), respectively. Previous studies have also revealed that the antioxidant capacity of kiwifruits is closely associated with their total polyphenols [2,3,8,9]. Notably, GA, CHA, NCHA, PA, ProcB1, ProcB2, ProcC1, QueR, and QueG showed significantly negative correlations with antioxidant capacity. Additionally, both CA and *p*-CA also exhibited strongly negative correlations with DPPH and OH radical scavenging ability. Overall, these results can provide sufficient evidence that DUKs possess plentiful natural antioxidants, thereby exhibiting great potential to be applied as functional ingredients in the food and pharmaceutical industries.

### 3.4. Comparison of Inhibitory Effect of Polyphenol-Enriched Extracts Obtained from Unripe, Mature, and Overripe Fruits of Red-Fleshed Kiwifruit Cultivars on α-Glucosidase

Numerous studies have revealed that the consumption of dietary polyphenols from fruits and vegetables can lower the risk of type 2 diabetes via inhibiting the enzymatic activities of both α-glucosidase and α-amylase [22,23]. In fact, kiwifruits exert remarkable inhibitory effects against α-glucosidase. Nevertheless, the inhibitory effect of unripe, mature, and overripe kiwifruits against α-glucosidase remains unclear. As displayed in Figure 3D, all tested samples exerted strong inhibitory effects on α-glucosidase. Obviously, the results showed a significant variation in the inhibitory effect on α-glucosidase of different kiwifruits harvested at the YK, MK1, and MK2 stages, with the order being YK > MK1 > MK2. In detail, the average IC_50_ values for α-glucosidase inhibition of thinned unripe, mature, and overripe kiwifruits were in the range of 17.8–457.0 μg/mL, and it could be clearly observed that the inhibitory effect of unripe kiwifruits on α-glucosidase was about 15.5 and 25.7 times stronger than that of mature and overripe kiwifruits, respectively. Furthermore, according to the correlation analysis as displayed in Figure 4, the IC_50_ values of the inhibitory effect on α-glucosidase showed significantly negative relationships to levels of TPC (r, −0.818), TFC (r, −0.723), and TPAC (r, −0.778), which was comparable to earlier studies [8,11,24]. In addition, PA, ProcB2, ProcB1, ProcC1, GA, CHA, and QueR displayed significantly negative relationships to the IC_50_ values of the inhibition effects on α-glucosidase, with r values in the range of −0.809 (ProcB2) to −0.736 (CHA), implying that ProcB2 was one of the most important compounds in the inhibitory effect on α-glucosidase. In fact, ProcB2 has been found as a strong inhibitor towards α-glucosidase in earlier studies [25,26]. Collectively, these results indicated that DUKs have good potential to be developed as natural inhibitors toward α-glucosidase in the functional food industry.

### 3.5. Comparison of Anti-Inflammatory Activity of Polyphenol-Enriched Extracts Obtained from Unripe, Mature, and Overripe Fruits of Red-Fleshed Kiwifruit Cultivars

Accumulating experimental results have shown that different species of kiwifruits have remarkable anti-inflammatory activity in vitro and in vivo [12,16,17]. Therefore, to exploit DUKs as potential functional ingredients, the in vitro anti-inflammatory activity of polyphenol-enriched extracts obtained from unripe, mature, and overripe fruits of red-fleshed kiwifruit cultivars were compared. Figure 3E shows the impacts of different unripe, mature, and overripe kiwifruits on the proliferation rate of RAW 264.7 cells. Notably, all tested samples had no cytotoxic effects on RAW 264.7 cells. In addition, as displayed in Figure 3F–H, all kiwifruit extracts could significantly inhibit the secretion of proinflammatory factors (NO, TNF-α and IL-6) from LPS-induced RAW 264.7 cells, implying that discarded unripe kiwifruits also possessed remarkable anti-inflammatory activity, similar to mature kiwifruits in earlier studies [27,28,29,30]. Obviously, the results showed a great variation in anti-inflammatory activity of different kiwifruits harvested at the YK, MK1, and MK2 stages, with the order being YK > MK1 > MK2. In detail, at a concentration of 100.00 µg/mL, the average inhibition rates of different kiwifruits harvested at the YK, MK1, and MK2 stages against the production of NO were about 48.13%, 41.99%, and 39.84, respectively. In addition, the average inhibition rates of different kiwifruits harvested at the YK, MK1, and MK2 stages against the secretion of TNF-α were about 58.81%, 49.09%, and 41.31%, respectively. Moreover, the average inhibition rates of different kiwifruits harvested at the YK, MK1, and MK2 stages against the secretion of IL-6 were about 58.34%, 45.56%, and 41.21%, respectively. These results indicated that discarded unripe kiwifruits exerted excellent in vitro anti-inflammatory activity. Moreover, as displayed in Figure 4, the levels of TPC and TPAC showed greatly positive relationships to the inhibitory rates of different kiwifruits against the secretion of IL-6 and TNF-α from LPS-stimulated RAW 264.7 cells, implying that total phenolics and total procyanidins could contribute to their anti-inflammatory activity [27,31,32]. Indeed, as displayed in Figure 4, ProcB1, ProcB2, ProcC1, PA, GA, CHA, and QueR showed obviously positive correlations with the inhibition rates of different kiwifruits against the release of IL-6 and TNF-α from LPS-stimulated RAW 264.7 cells, and QueG, FA, CA, NCHA, and PA exhibited notably positive correlations with the inhibition rates of different kiwifruits against the production of NO. In fact, several studies have demonstrated that these procyanidins (e.g., ProcB1, ProcB2, and ProcC1) [33,34,35] and phenolic acids (FA, GA, CA, CHA, and NCHA) [36,37,38,39] possess excellent anti-inflammatory activity. Overall, these results suggest that DUKs can be developed as functional ingredients for the amelioration of chronic inflammatory diseases.

## 4. Conclusions

DUKs are regarded as the major agro-byproducts in the production of kiwifruits. Due to the limited knowledge about the phytochemicals and biological function of these agro-byproducts, their current utilizations in the food industry remain scarce. Hence, to improve their development and applications in the food or functional food industries, the phenolic compounds and health-promoting effects of discarded unripe, mature, and overripe kiwifruits were studied. Our findings revealed that DUKs possessed greatly higher contents of valuable phenolic compounds than mature and overripe kiwifruits, with ECN, ProcB2, ProcB1, and NCHA observed as the predominate polyphenols. In addition, compared with mature and overripe kiwifruits, DUKs exerted much stronger antioxidant capacity, inhibitory effect on α-glucosidase, and anti-inflammatory activity, which were probably attributed to their higher contents of phenolic compounds, such as GA, CHA, NCHA, PA, ProcB1, ProcB2, ProcC1, and QueR. Overall, our findings can provide sufficient evidence for the development and utilization of DUKs in the food/functional food industry. Nevertheless, we did not study anthocyanins in DUKs, which was the limitation of the present study. These important compounds can be investigated in future to further exploit the potential applications of DUKs.

## Figures and Tables

**Table 1:** Tentative identification of phenolic compounds in polyphenol-enriched extracts obtained from red-fleshed kiwifruit cultivars with different maturities by LC-Q-TOF-MS.

NO.	Identified Compounds	Formula	RT (min)	Molar Mass	Observed *m*/*z*	Score	Error (ppm)	Kiwifruit Extracts
1	Quinic acid	C_7_H_12_O_6_	0.54	192.0634	191.0561	99.90	0.09	a–f ^##^
2	Malic acid	C_4_H_6_O_5_	0.57	134.0215	133.0142	87.46	−0.11	a–f ^##^
3	Citric acid	C_6_H_8_O_7_	0.64	192.0270	191.0196	99.55	−0.10	a–f ^##^
4	Fumaric acid	C_4_H_4_O_4_	0.69	116.0110	115.0038	87.57	0.70	a–f ^##^
5	Succinic acid	C_4_H_6_O_4_	0.77	118.0271	117.0198	98.34	3.76	a–f ^##^
6	*p*-Coumaric acid-hexose	C_15_H_19_O_9_	1.16	343.1026	342.0953	87.30	−0.94	a, b, c ^##^
7	Gallic acid	C_7_H_6_O_5_	1.36	170.0233	169.0161	80.64	2.38	a–f ^###^
8	Protocatechuic acid	C_7_H_6_O_4_	1.62	154.0266	153.0193	98.83	−0.02	a–i ^###^
9	Protocatechuic acid-O-hexoside	C_13_H_16_O_9_	1.72	316.0802	315.073	97.28	2.35	a–f ^##^
10	Dihydroxyphenylpropionic acid	C_9_H_10_O_4_	1.80	182.0584	181.0511	84.86	2.44	a, b, c ^#^
11	Aconitic acid	C_6_H_6_O_6_	2.44	174.0169	173.0095	83.81	2.48	a, b, c ^#^
12	Cinnamic acid	C_9_H_8_O_2_	2.97	148.0527	147.0456	81.02	1.58	a–f ^##^
13	Neochlorogenic acid	C_16_H_18_O_9_	3.30	354.0958	353.0886	98.20	2.13	a–i ^###^
14	Procyanidin B1	C_30_H_26_O_12_	3.54	578.1437	577.137	94.68	2.21	a–i ^###^
15	Catechin	C_15_H_14_O_6_	4.78	290.0796	289.0723	98.65	2.04	a–i ^###^
16	Chlorogenic acid	C_16_H_18_O_9_	5.42	354.0958	353.0886	98.20	2.13	a–f ^###^
17	Vanillic acid	C_8_H_8_O_4_	6.13	168.0421	167.0348	98.98	−0.94	a–f ^##^
18	Caffeic acid	C_9_H_8_O_4_	6.89	180.0423	179.035	99.77	0.45	a, b, d, e, g, h ^###^
19	Syringic acid	C_9_H_10_O_5_	7.52	198.0532	197.0459	98.53	1.88	a–f ^##^
20	3-*p*-Coumaroylquinic acid	C_16_H_18_O_8_	9.82	338.1007	337.0934	83.43	1.47	a–f ^##^
21	Caffeic acid-O-hexoside	C_15_H_18_O_9_	10.10	342.0965	341.0894	88.78	4.16	a–f ^##^
22	Procyanidin B2	C_30_H_26_O_12_	10.77	578.1437	577.137	94.68	2.21	a–i ^###^
23	Epicatechin	C_15_H_14_O_6_	12.22	290.0796	289.0723	98.65	2.04	a–i ^###^
24	*p*-Coumaric acid	C_9_H_8_O_3_	12.88	164.0479	163.0407	85.21	3.52	a, b, d, e, g, h ^###^
25	Ferulic acid	C_10_H_10_O_4_	15.89	194.0581	193.0508	85.60	0.79	a, b, d, e, g, h ^###^
26	Quercetin 3-O-glucoside	C_21_H_20_O_12_	16.54	464.0952	463.0883	84.92	0.75	a, b, d, e, g, h ^###^
27	Quercetin 3-O-rhamnoside	C_21_H_20_O_11_	17.20	448.1011	447.0941	85.65	0.74	a–i ^###^
28	Procyanidin C1	C_45_H_38_O_18_	18.17	866.2057	865.1985	99.59	−0.09	a–i ^##^
29	Apigenin	C_15_H_10_O_5_	23.68	270.0536	269.0462	80.85	2.83	a–f ^#^
30	Quercetin-3-O-robinobioside	C_27_H_30_O_16_	24.78	610.1539	609.1466	98.87	0.90	a, b, c ^#^
31	Quercetin	C_15_H_10_O_7_	27.14	302.0414	301.0342	93.96	−4.08	a–i ^##^
32	Kaempferol	C_15_H_10_O_6_	28.60	286.0480	285.0406	84.92	2.13	a–f ^##^
33	Isovitexin	C_21_H_20_O_10_	29.87	432.1060	431.0988	98.38	0.90	a, b, c ^#^

^#^ Compared with database; ^##^ Compared with database and the literature; ^###^ Compared with database, the literature. and authentic phenolic standards; a–i stand for polyphenol-enriched extracts obtained from ‘Hongao’ (HA), ‘Hongshi’ (HS), and ‘Hongyang’ (HY) kiwifruit cultivars with different maturation stages, including the immature stage (YK), mature stage (MK1), and overmature stage (MK2), which were coded as HA-YK, HS-YK, HY-YK, HA-MK1, HS-MK1, HY-MK1, HA-MK2, HS-MK2, and HY-MK2, respectively.

**Table 2:** Contents of major phenolic compounds in polyphenol-enriched extracts obtained from red-fleshed kiwifruit cultivars with different maturities.

Compounds	HA-YK	HA-MK1	HA-MK2	HS-YK	HS-MK1	HS-MK2	HY-YK	HY-MK1	HY-MK2
GA	622.8 ± 19.5 ^b^	17.7 ± 0.8 ^c^	N.D.	685.3 ± 15.9 ^a^	21.2 ± 0.7 ^c^	N.D.	707.7 ± 23.6 ^a^	33.0 ± 1.4 ^c^	N.D.
PA	823.0 ± 40.9 ^b^	20.2 ± 1.1 ^d^	16.6 ± 0.6 ^d^	1168.4 ± 39.7 ^a^	95.9 ± 1.3 ^c^	27.1 ± 0.9 ^d^	852.1 ± 35.9 ^b^	72.1 ± 1.0 ^cd^	20.2 ± 0.9 ^d^
NCHA	5929.3 ± 127.1 ^a^	207.1 ± 7.4 ^e^	170.9 ± 5.9 ^e^	5829.6 ± 203.7 ^b^	382.5 ± 12.8 ^d^	200.7 ± 10.3 ^e^	1157.4 ± 45.7 ^c^	229.0 ± 11.6 ^e^	146.9 ± 5.7 ^e^
ProcB1	3554.6 ± 141.6 ^b^	523.6 ± 23.5 ^d^	458.8 ± 16.0 ^d^	2408.5 ± 152.0 ^c^	327.3 ± 12.7 ^e^	155.9 ± 8.1 ^f^	3708.5 ± 153.2 ^a^	477.3 ± 15.3 ^d^	216.0 ± 7.8 ^f^
CN	832.8 ± 12.4 ^b^	536.6 ± 18.3 ^d^	195.1 ± 7.2 ^f^	1142.6 ± 15.2 ^a^	612.5 ± 23.7 ^c^	305.4 ± 15.2 ^e^	N.D.	N.D.	N.D.
CHA	249.4 ± 9.6 ^a^	11.3 ± 0.5 ^d^	N.D.	176.0 ± 6.3 ^b^	5.9 ± 0.1 ^e^	N.D.	92.7 ± 1.1 ^c^	3.2 ± 0.1 ^e^	N.D.
CA	63.2 ± 1.3 ^a^	11.5 ± 0.6 ^c^	8.7 ± 0.2 ^c^	63.3 ± 2.4 ^a^	17.1 ± 0.4 ^b^	4.1 ± 0.6 ^d^	N.D.	N.D.	N.D.
ProcB2	8583.1 ± 281.3 ^a^	1933.3 ± 87.2 ^d^	1742.2 ± 28.6 ^de^	6901.8 ± 227.6 ^b^	1547.6 ± 58.6 ^e^	1260.3 ± 67.6 ^f^	6459.2 ± 259.4 ^c^	1563.4 ± 59.3 ^e^	1242.5 ± 13.9 ^f^
ECN	5388.7 ± 103.6 ^a^	1638.9 ± 129.6 ^c^	877.3 ± 11.2 ^de^	2482.2 ± 133.5 ^b^	1095.8 ± 49.1 ^d^	702.8 ± 13.3 ^e^	210.6 ± 7.3 ^f^	87.3 ± 4.9 ^g^	70.3 ± 2.7 ^g^
*p*-CA	186.7 ± 6.9 ^a^	7.7 ± 0.2 ^c^	6.0 ± 0.2 ^c^	165.7 ± 3.2 ^b^	7.5 ± 0.2 ^c^	6.2 ± 0.2 ^c^	N.D.	N.D.	N.D.
FA	12.8 ± 0.4 ^b^	3.1 ± 0.1 ^d^	2.2 ± 0.1 ^e^	22.3 ± 1.0 ^a^	6.6 ± 0.2 ^c^	3.7 ± 0.2 ^d^	N.D.	N.D.	N.D.
QueG	825.9 ± 33.9 ^a^	69.8 ± 0.3 ^c^	8.6 ± 0.3 ^e^	794.1 ± 9.6 ^b^	49.7 ± 1.1 ^d^	7.6 ± 0.3 ^e^	N.D.	N.D.	N.D.
QueR	574.8 ± 1.4 ^c^	28.1 ± 0.2 ^f^	7.1 ± 0.1 ^g^	636.9 ± 5.8 ^b^	38.1 ± 1.4 ^e^	8.0 ± 0.2 ^g^	658.4 ± 5.0 ^a^	49.0 ± 1.3 ^d^	8.1 ± 0.5 ^g^
ProcC1	405.8 ± 6.8 ^a^	128.3 ± 3.2 ^d^	29.3 ± 0.9 ^f^	341.2 ± 7.6 ^b^	136.1 ± 1.2 ^d^	92.0 ± 1.1 ^e^	418.3 ± 10.6 ^a^	197.9 ± 3.5 ^c^	81.5 ± 2.5 ^e^
Total content (μg/g DW)	27,918.0 ± 963.6 ^a^	5137.1 ± 239.5 ^d^	3522.9 ± 143.1 ^ef^	23,718.2 ± 452.7 ^b^	4405.1 ± 168.8 ^e^	2973.8 ± 154.7 ^f^	14,265.0 ± 505.4 ^c^	2934.8 ± 74.1 ^f^	2031.5 ± 102.8 ^g^

HA-YK, HS-YK, HY-YK, HA-MK1, HS-MK1, HY-MK1, HA-MK2, HS-MK2, and HY-MK2 indicate polyphenol-enriched extracts obtained from ‘Hongao’ (HA), ‘Hongshi’ (HS), and ‘Hongyang’ (HY) kiwifruit cultivars at different maturation stages, including the immature stage (YK), mature stage (MK1), and overmature stage (MK2); GA, gallic acid; PA, protocatechuic acid; NCHA, neochlorogenic acid; ProcB1, procyanidin B1; CN, catechin; CHA, chlorogenic acid; CA, caffeic acid; ProcB2, procyanidin B2; ECN, epicatechin; FA, ferulic acid; *p*-CA, *p*-coumaric acid; QueG, quercetin 3-O-glucoside; QueR, quercetin 3-O-rhamnoside; ProcC1, procyanidin C1; different letters in the same column indicate significant differences at *p* < 0.05; N.D. means not detected or that the concentration was too low to be quantified.

## Data Availability

The original contributions presented in the study are included in the article/Supplementary Materials, further inquiries can be directed to the corresponding authors.

## Supplementary Materials

The following supporting information can be downloaded at: https://www.mdpi.com/article/10.3390/foods13182860/s1, Section S.1: Preparation of polyphenol-enriched extracts from different kiwifruits; Section S.2: Determination of total polyphenols in polyphenol-enriched extracts obtained from different kiwifruits; Section S.3: Qualitative analysis of phenolic compounds in different polyphenol-enriched extracts by LC-Q-TOF-MS analysis; Section S.4: Quantitative analysis of major phenolic compounds in different polyphenol-enriched extracts by HPLC analysis; Section S.5: Evaluation of biological activities of polyphenol-enriched extracts obtained from different kiwifruits; Table S1: Calibration data for fourteen phenolic compounds.
