# Supplementary material for: Characterization, Antioxidant Capacity, and Anti-Inflammatory Activity of Polyphenol-Enriched Extracts Obtained from Unripe, Mature, and Overripe Fruits of Red-Fleshed Kiwifruit Cultivars
Source: Foods. 2024 Sep 10;13(18):2860. doi: 10.3390/foods13182860 (PMC11430867; doi:10.3390/foods13182860)
Supplement: Supplementary file 1 [file foods-13-02860-s001.zip › foods-3183858-supplementary.pdf]

Supplementary Materials

Characterization, Antioxidant Capacity, and Anti-Inflammatory Activity of Polyphenol-Enriched Extracts Obtained from Unripe, Mature, and Overripe Fruits of Red-Fleshed Kiwifruit Cultivars

Qian-Ni Yang [1,2], Wen Deng [1,2], Ding-Tao Wu [2,*], Jie Li [3], Hong-Yan Liu [3], Hui-Ling Yan [1], Kui Du [4], Yi-Chen Hu [1], Liang Zou [1] and Jing-Wei Huang [1,*]

[1] Key Laboratory of Coarse Cereal Processing of Ministry of Agriculture and Rural Affairs, School of Food and Biological Engineering, Chengdu University, Chengdu 610106, China

[2] Institute for Advanced Study, Chengdu University, Chengdu 610106, China

[3] Research Center for Plants and Human Health, Institute of Urban Agriculture, Chinese Academy of Agri-cultural Sciences, National Agricultural Science and Technology Center, Chengdu 610213, China

[4] China-New Zealand Belt and Road Joint Laboratory on Kiwifruit, Kiwifruit Breeding and Utilization Key Laboratory of Sichuan Province, Sichuan Provincial Academy of Natural Resource Sciences, Chengdu 610015, China

[*] Correspondence: wudingtao@cdu.edu.cn (D.-T.W.); huangjingwei@cdu.edu.cn (J.-W.H.)

Section S.1 Preparation of polyphenol-enriched extracts from different kiwifruits

Polyphenol-enriched extracts from different discarded immature kiwifruits, mature kiwifruits, and overmature kiwifruits were prepared by ultrasound-assisted deep eutectic solvent extraction (UADEE). In detail, the deep eutectic solvent (DES) was formulated by mixing choline chloride and glycerol in a molar ratio of 1: 2, followed by the addition of 32% (*v/v*) of water under gentle stirring. The mixture was then sonicated by an ultrasonic cleaning cell until a homogenous solution was obtained. 1.0 g of each kiwifruit powder was mixed with 50 mL of DES extraction solvent, and extracted using a JY92-IIN ultrasonic processor (Ningbo Scientz Biotechnology Co., Ltd., Ningbo, China). The ultrasonic extraction power and ultrasonic extraction time were set as 450 W and 23 min, respectively. The polyphenol-enriched extract was then subjected to centrifugation (6000 × g, 4 °C, 15 min), and the supernatant was filtered through an organic filter membrane. The extracted supernatant was stored at 4 °C for further analysis.

Section S.2 Determination of total polyphenols in polyphenol-enriched extracts from different kiwifruits

The contents of total polyphenols, including total phenolic content (TPC), total flavonoid content (TFC), and total procyanidin content (TPAC), were determined by colorimetric methods. In detail, the levels of TPC in different discarded immature kiwifruits, mature kiwifruits, and overmature kiwifruits were determined using a modified Folin-Ciocalteu colorimetric method. A suitably diluted kiwifruit extract (100 μL) was mixed with 500 μL of Folin-Ciocalteu working solution (0.2 M) and reacted at

room temperature for 10 min. Afterward, 500 μL of sodium carbonate solution (20%, *w/v*) was added, and the mixture was incubated in the dark for 60 min at room temperature. The absorbance of the mixture was measured at 760 nm with gallic acid as a standard. TPC was expressed as milligram gallic acid equivalent per gram kiwifruit dry weight (mg GAE/g DW).

The levels of TFC in different discarded immature kiwifruits, mature kiwifruits, and overmature kiwifruits were quantified using the $AlCl_3$-based colorimetric method. Briefly, the diluted kiwifruit extract (100 μL) was mixed with 30 μL of $NaNO_2$ (5%, *w/v*) solution and left for 6 min. Then, 30 μL of Al $(NO_3)_3$ (10%, *w/v*) solution was added, and the mixture was reacted for an additional 6 min. Afterward, 400 μL of NaOH (4%, *w/v*) was added, and then the mixture was kept in the dark for 25 min. Finally, the absorbance of the mixture was measured at 510 nm. Rutin was served as a standard for the quantification. TFC was expressed as milligram rutin equivalent per gram kiwifruit dry weight (mg RE/g DW).

The levels of TPAC in different discarded immature kiwifruits, mature kiwifruits, and overmature kiwifruits were quantified using a modified vanillin-sulfuric acid colorimetric method. In brief, A mixture of kiwifruit extract (300 μL), 5% methanolic vanillin solution (750 μL), and 20% methanolic sulfuric acid solution (750 μL) was shaken and heated at 30 °C for 20 min in the dark. The absorbance of the mixture was measured at 500 nm with catechin as a standard. TPAC was expressed as milligram catechin equivalent per gram kiwifruit dry weight (mg CE/g DW).

Section S.3 Qualitative analysis of phenolic compounds in different polyphenol-

enriched extracts by LC-Q-TOF-MS analysis

Individual phenolic compounds in different polyphenol-enriched extracts from discarded immature kiwifruits, mature kiwifruits, and overmature kiwifruits were analyzed by LC-Q-TOF-MS (Agilent 6545 Q-TOF-MS, Agilent Technologies, Santa Clara, CA, USA). An Agilent ZORBAX RRHD Eclipse Plus C18 column (2.1 × 50 mm, 1.8 μm) was utilized for the separation of kiwifruit extracts. The mobile phase was consisted of 0.5% formic acid aqueous solution (solvent A) and acetonitrile (solvent B). The column was eluted with a gradient of 5% B (0-5 min), 5-7% B (5-10 min), 7-10% B (10-20 min), 10-30% B (20-40 min), 30-95% B (40-45 min), 95% B (45-50 min), 95-5% B (50-51 min), and 5% B (51-56 min). The column temperature, flow rate, and injection volume were 30 °C, 0.3 mL/min, and 5 μL, respectively. The high-resolution Q-TOF-MS was operated in the negative ion mode, and scanned in a mass range of *m/z* 100 – 1000. The capillary voltage, drying gas temperature, drying gas flow, and nebulizing gas pressure were set as +4.0 kV, 350 ℃, 10.0 L/min, and 40.0 psi, respectively. Data analysis was conducted using Agilent Qualitative Analysis 10.0 software (Agilent Technologies, Santa Clara, CA, USA) and Agilent PCDL Manager B. 08.00 software (Agilent Technologies, Santa Clara, CA, USA). Parent ions were cross-referenced with TCM-database (Agilent Technologies, Santa Clara, CA, USA) and literatures as well as several authentic standards to identify phenolic compounds in different polyphenol-enriched extracts.

Section S.4 Quantitative analysis of major phenolic compounds in different polyphenol-enriched extracts by HPLC analysis

The major phenolic compounds in polyphenol-enriched extracts from different discarded immature kiwifruits, mature kiwifruits, and overmature kiwifruits were measured using an Agilent 1260 HPLC system (Agilent Technologies, Santa Clara, CA, USA). The chromatographic separation was performed at 25 °C using a ZORBAX Eclipse XDB-C18 column (250 mm × 4.6 mm, 5 µm, Agilent Technologies, Santa Clara, CA, USA). The mobile phase was consisted of solvent A (0.5% of acetic acid aqueous solution) and solvent B (acetonitrile). Samples were eluted as follows: 0 min, 5% B; 5 min, 5% B; 50 min, 5-20% B; 70 min, 20-70% B; 72 min, 70-5% B; and 72-77 min, 5% B. The flow rate was 0.8 mL/min, and the injection volume was 20 µL for all samples. Hydroxybenzoic acid and flavanols were determined at 280 nm, hydroxycinnamic acids were measured at 320 nm, and flavonols were detected at 360 nm. In this study, fourteen commercially available phenolic standards, including six phenolic acids (gallic acid, caffeic acid, ferulic acid, *p*-coumaric acid, chlorogenic acid, and neochlorogenic acid), six flavanols (protocatechuic acid, catechin, epicatechin, procyanidin B1, procyanidin B2, and procyanidin C1), and two flavonols (quercetin 3-O-glucoside and quercetin 3-O-rhamnoside), were quantified, and their calibration curves were shown in Table S1. The levels of individual phenolic compounds in different polyphenol-enriched extracts were expressed as microgram per gram kiwifruit dry weight (µg/g DW).

Table S1. Calibration data for fourteen phenolic compounds

Compounds	Regression equation	R^2	Linear range (µg/mL)
Gallic acid	y = 13.2193x – 37.637	R^2 = 0.9953	2.8 – 35.1
Protocatechuic acid	y = 15.774x – 41.2134	R^2 = 0.9967	2.8 – 35.1
Neochlorogenic acid	y = 42.504x – 137.738	R^2 = 0.9992	3.1 – 167.9
Procyanidin B1	y = 10.399x – 5.277	R^2 = 0.9988	3.1 – 167.9
Catechin	y = 8.263x – 57.556	R^2 = 0.9961	1.1 – 54.1
Chlorogenic acid	y = 21.2831x – 87.637	R^2 = 0.9964	4.1 – 36.4
Caffeic acid	y = 156.36x – 78.135	R^2 = 0.9977	2.8 – 35.1
Procyanidin B2	y = 8.53x – 13.256	R^2 = 0.9996	18.8 – 173.9
Epicatechin	y =12.664x – 26.514	R^2 = 0.9982	18.8 – 173.9
p-Coumaric acid	y = 189.231x – 135.783	R^2 = 0.9968	2.8 – 35.1
Ferulic acid	y = 219.511x – 38.579	R^2 = 0.9953	2.8 – 35.1
Quercetin 3-O-glucoside	y = 37.801x – 21.6951	R^2 = 0.9967	1.1 – 54.1
Quercetin 3-O-rhamnoside	y = 27.054x – 82.157	R^2 = 0.9952	1.1 – 54.1
Procyanidin C1	y = 187.514x – 655.166	R^2 = 0.9995	1.1 – 54.1

Section S.5 Evaluation of biological functions of polyphenol-enriched extracts from different kiwifruits

S.5.1 Assessment of antioxidant capacities of different polyphenol-enriched extracts

To systematically understand the differences in antioxidant capacities of polyphenol-enriched extracts from different discarded immature kiwifruits, mature kiwifruits, and overmature kiwifruits, the ABTS radical scavenging ability assay, DPPH radical scavenging ability assay, and hydroxyl radical (OH) scavenging ability assay were carried out in this study. For the determination of ABTS radical scavenging ability, the ABTS radical solution was generated by the interaction of 7 mM ABTS solution and 2.45 mM aqueous potassium persulfate at room temperature for at least 16 h in the dark. The ABTS radical solution was then diluted with phosphate buffer (0.2 M, pH 7.4)

to an absorbance of 0.780 ± 0.05 at 734 nm. Afterward, 200 μL of ABTS radical working solution was mixed with 20 μL of each kiwifruit extract at five different concentrations or phosphate buffer as a negative control in a 96-well microplate to react at 30 °C for 20 min. The absorbance of the mixture was measured at 734 nm, and Trolox was used as a standard. The IC_{50} values were calculated by establishing a logarithmic regression. In addition, for the determination of DPPH radical scavenging ability, 25 μL of each kiwifruit extract at five different concentrations or methanol as a negative control was added to 200 μL of DPPH solution (0.35 mM) in a 96-well microplate. The mixed solution was shaken and incubated at room temperature for 30 min. Finally, the absorbance of the mixture was measured at 517 nm with a blank contain-only DPPH solution and methanol. Trolox was also used as a standard. The IC_{50} values were calculated by establishing a logarithmic regression. Furthermore, for the determination of OH radical scavenging ability, 100 μL of each kiwifruit extract was mixed with 100 μL of $FeSO_4$ (2 mM) and 100 μL of salicylic acid-ethanol solution (6 mM). Afterward, 100 μL of H_2O_2 (6 mM) was added to the mixture and then incubated for 30 min at 37 °C in a water bath. The absorbance of the mixture was measured at 510 nm, and the IC_{50} values were calculated by establishing a logarithmic regression.

S.5.2 Assessment of inhibitory effects of different polyphenol-enriched extracts against α-glucosidase

To understand the differences in potential anti-diabetic effects of polyphenol-enriched extracts from different discarded immature kiwifruits, mature kiwifruits, and overmature kiwifruits, their inhibitory effects against α-glucosidase were measured. In

brief, 100 μL of each kiwifruit extract at five different concentrations was mixed with 100 μL of α-glucosidase (0.5 U/mL, pH 6.8) for 10 min at 37 °C. Subsequently, 25 μL of *p*-nitrophenyl-α-D-glucopyranoside solution (4 mM, pH 6.8) was added, and then the mixture was further incubated for 20 min at 37 °C under dark conditions. The absorbance of the mixture was measured at 405 nm, and acarbose was employed as a positive control. The IC_{50} values of different polyphenol-enriched extracts against α-glucosidase were expressed as microgram kiwifruit dry weight per milliliter (μg/mL).

S.5.3 Assessment of anti-inflammatory activities of different polyphenol-enriched extracts

To understand the differences in potential anti-inflammatory activities of polyphenol-enriched extracts from different discarded immature kiwifruits, mature kiwifruits, and overmature kiwifruits, the lipopolysaccharide (LPS)-induced RAW 264.7 cell model was carried out to evaluate their *in vitro* anti-inflammatory activities. In brief, the impact of each kiwifruit extract on the cell viability of RAW 264.7 cells was evaluated by the MTT method, and different concentrations (25-100 μg kiwifruit dry weight/mL, μg/mL) of each kiwifruit extract were tested. In addition, the RAW 264.7 macrophages were cultivated overnight in 96-well plates at 37 °C with 5% CO_2. The culture medium was then replaced with LPS (1 μg/mL) in a volume of 100 μL per well, except for the blank group. After incubating for 24 h, various concentrations (25-100 μg/mL) of each kiwifruit extract were added and incubated for an additional 24 h. The supernatant (50.0 μL) was mixed with Griess I (50.0 μL) and Griess II (50.0 μL) reagents at room temperature, and the absorbance was measured at 540 nm. $NaNO_2$ was

employed as a reference standard to determine the concentration of NO. The levels of cytokines interleukin-6 (IL-6) and tumor necrosis factor-alpha (TNF-α) in the supernatant were measured by ELISA kits based on the manufacturer's procedures (Elabscience, Wuhan, China).
